# Senescence risk score: a multifaceted prognostic tool predicting outcomes, stemness, and immune responses in colorectal cancer

**DOI:** 10.3389/fimmu.2023.1265911

**Published:** 2023-09-26

**Authors:** Xiaojun Zhang, Yilan Huang, Qian Li, Yiqing Zhong, Yuanzhou Zhang, Jingying Hu, Rui Liu, Xiaoying Luo

**Affiliations:** ^1^ State Key Laboratory of Systems Medicine for Cancer, Shanghai Cancer Institute, Renji Hospital, Shanghai Jiao Tong University School of Medicine, Shanghai, China; ^2^ Key Laboratory of Carcinogenesis and Cancer Invasion, Ministry of Education, Fudan University, Shanghai, China; ^3^ Department of Oncology, Shanghai Jiao Tong University Affiliated Sixth People’s Hospital, Shanghai, China; ^4^ Department of Gastrointestinal Surgery, Renji Hospital, School of Medicine, Shanghai Jiao Tong University, Shanghai, China

**Keywords:** cellular senescence, colorectal cancer, prognosis, tumor immune microenvironment, cell stemness, machine learning

## Abstract

Colorectal cancer (CRC) remains a primary cause of cancer mortality globally, necessitating precise prognostic indicators for effective clinical management. Our study introduces the Senescence Risk Score (SRRS), based on several senescence-related genes (SRGs), a potent prognostic tool designed to measure cellular senescence in CRC. The higher SRRS predicts a poorer prognosis, providing a novel and efficient approach to patient stratification. Notably, we found that SRRS correlates with methylation and mutation variations, and increased immune infiltration in the tumor microenvironment, thus revealing potential therapeutic targets. We also discovered an inverse relationship between SRRS and cell stemness, which could have significant implications for cancer treatment strategies. Utilizing bioinformatics resources and machine learning, we identified LIMK1 and WRN as key genes associated with SRRS, further enhancing its prognostic value. Importantly, the modulation of these genes significantly impacts cellular senescence, proliferation, and stemness in CRC cells. In summary, our development of SRRS offers a powerful tool for CRC prognosis and paves the way for novel therapeutic strategies, underscoring its potential in transforming CRC patient management.

## 1 Introduction

Globally, colorectal cancer (CRC) ranks as the third major cause of freshly diagnosed cancer and the second major cause of cancer-related deaths according to GLOBOCAN 2020 estimates (1). Its insidious onset often results in a late-stage diagnosis, during which time the disease has typically progressed significantly. Despite significant advancements in targeted therapy and immunotherapy, the prognosis for CRC remains gloomy, mainly due to the inability to precisely stratify patients and limited treatment options (2, 3). Hence, it is imperative to prioritize the identification and characterization of molecular subtypes of CRC in order to facilitate more accurate and targeted interventions.

A growing body of research has shed light on the dichotomous role of cellular senescence in CRC tumorigenesis and development (4–9). Cellular senescence is thought to prevent tumorigenesis at early stages (5, 10). However, persist standing senescent cells tend to be important contributors of the pro-tumorigenic effects, mainly through the senescence associated secretory phenotype (SASP), as they evolve over time (11). Thus, senescence is beneficial for tumor clearance in a short term, but may cause long-term disadvantages and ultimately lead to tumor progression, which holds the potential to be leveraged therapeutically if its adverse effects can be well addressed. Despite all this, quantifying levels of cellular senescence is challenging due to the lack of universal and specific markers (12). As a result, there is an urgent need to develop reliable methods to evaluate cellular senescence levels.

Recently, there has been a surge of interest in identifying senescence characteristics by combining with multiple transcriptional profiles of senescent cells (13–15). Accurately characterizing the level of cellular senescence in cancer patients is a complex task. Additionally, given the inconsistent manifestation of cellular senescence in different stages of disease progression, translating senescence-related mechanisms into clinical outcomes presents a challenge (16, 17). Therefore, identifying the level of cellular senescence with clinical relevance may provide potential biomarkers for prognosis prediction and therapy guidance. In this study, we propose a Senescence Risk Score (SRRS) based on several senescence-related genes (SRGs). This SRRS allows us to stratify CRC patients and assess the effect on prognosis. In addition, we evaluate the potential of SRRS in predicting immune treatment response and cell stemness in CRC patients. Upon defining the SRRS, we identify potential chemotherapeutic drugs and, through employing machine learning, delineate two pivotal genes within the SRRS model and validate their reliability. These findings unveil the intricate interplay of cellular senescence in CRC and present novel avenues for the development of therapeutic strategies targeting cellular senescence (18).

## 2 Materials and methods

### 2.1 Data and resources

Somatic mutation data (mutation annotation format), RNA-seq data (transcripts per million), and associated clinical data for CRC (589 samples) were downloaded from the TCGA (The Cancer Genome Atlas) database using the R package TCGAbiolinks (19) (v. 2.25.3). Persistent mutations and additional mutation data were collected from previously published literatures (20) (**Supplemental Table 1**). Expression profiles of immunotherapy cohorts were retrieved through accession numbers (2016_Ascierto, 2017_Ascierto, 2017_Riaz, 2017_Snyder, 2018_Auslander, 2020_Cho). Information from two CRC validation cohorts was downloaded from GSE39084 (Gene Expression Omnibus (GEO) database, https://www.ncbi.nlm.nih.gov/geo/) and GSE38832. Single-cell RNA-seq data were collected from GSE188711. The 279 senescence-related genes were collected from the CellAge database (http://genomics.senescence.info/cells) (14). We collected previously curated gene sets associated with senescence (**Supplemental Table 2**) and stemness (**Supplemental Table 3**) from the published literature (14, 21–23).

### 2.2 SRRS score calculation

By performing Cox analysis to screen for prognosis-related genes in TCGA-CRC(CRC sample with expression data from TCGA) (P < 0.05), we identified 14 beneficial genes (bene-SRGs) and 16 detrimental genes (detri-SRGs). The bene-SRGs include BRCA1, CHEK1, CSNK1A1, CXCL1, ETS2, GLB1, MAD2L1, NEK4, PBRM1, PDPK1, PTTG1, SIK1, SRSF1, and WRN. The detri-SRGs include BCL6, CDKN2A, FASTK, IGFBP3, IRF7, LIMK1, MAPK12, MECP2, NOTCH3, NOX4, PCGF2, SIN3B, SIX1, SNAI1, UBTD1, and YPEL3. Using the gene expression data of these genes, we established the SRRS model to indicate the level of senescence risk in CRC. Single-sample gene set enrichment analysis (ssGSEA) from the ‘GSVA’ (v.1.46.0) R package (24) was utilized to calculate the enrichment scores of bene-SRGs and detri-SRGs. The SRRS for tissue samples, cancer cell lines, and single cells was calculated as the difference between the ssGSEA scores of detri-SRGs and bene-SRGs.

### 2.3 PCA and t-SNE analysis

Principal Component Analysis (25) (PCA) and t-Distributed Stochastic Neighbor Embedding (26) (t-SNE) were utilized for dimensionality reduction analysis, enabling the visualization of the segregation patterns of detri-SRGS and bene-SRGs in TCGA-CRC.

### 2.4 Survival analysis

The survival differences between the two groups were assessed through Kaplan-Meier survival curves. The log-rank test was employed to determine significant differences, with a p-value < 0.05 considered statistically significant. The survival analyses were conducted using the R packages ‘survival’ (v.3.4-0) and ‘survminer’ (v.0.4.9).

### 2.5 Construction of predictive nomogram

To identify independent prognostic indicators for CRC patients, univariate and multivariate Cox proportional hazards regression models were conducted using the ‘survival’ R package. A clinical characteristic with a p-value less than 0.05 was considered significantly associated with the survival of CRC patients. The hazard ratio (HR) with a 95% confidence interval (CI) and corresponding p-values were visualized using the ‘ggforest’ function. Clinical stage, age, and SRRS were utilized to construct a predictive nomogram, which allowed for a quantitative assessment of the prognostic risk for CRC patients. Calibration curves at 1, 3 and 5 years were drawn to evaluate the predictive capability of the nomogram. Additionally, the decision curve analysis (DCA) for 1,3 and 5 years was employed to assess the net clinical benefits of using SRRS and the predictive nomogram.

### 2.6 Identification of DNA methylation-driven genes

The ‘minfi’ (v.1.44.0) R package (27) was utilized for reading and normalizing DNA methylation data. The ‘limma’ (v.3.54.0) R package (28) was applied for the analysis to identify differentially methylated sites. Furthermore, the differentially methylated sites were mapped to genes using the Wannovar website (http://wannovar.wglab.org/).

### 2.7 Enrichment analysis and immune landscape of SRRS subtypes

The differential analysis between two SRRS subtypes was conducted using the ‘limma’ R package. We used the ‘clusterProfiler’ (v.4.6.0) R package (29) to implement the Gene Set Enrichment Analysis (GSEA), using all genes and their log2 FC (fold changes) as a basis. For the enrichment analysis, gene sets of ‘c2.cp.kegg.v7.2.symbols’ were downloaded from the Molecular Signatures Database (MSigDB) and processed through the GSEA. Additionally, Gene Ontology (30) (GO) and Kyoto Encyclopedia of Genes and Genomes (31) (KEGG) enrichment analyses were performed, with a False Discovery Rate (FDR) of <0.05 indicating significant enrichment. Furthermore, FDR <0.05 was set to denote significant differences within the KEGG and GO analyses.To assess the extent of immune cell infiltration, the ‘Xcell’ (32) (v.1.1.0) and ‘MCPcounter’ (33) (v.1.2.0) algorithms were employed to quantify immune cell signatures across TCGA-CRC. The ‘estimate’ (34) (v.1.0.13) R package was used to compute the IMMUNE and ESTIMATE scores of CRC patients.To better understand the immune characteristics of the SRRS groups, we compared the gene expression of major histocompatibility complexes and T-cell stimulators across different clusters. We also utilized gene expression data and corresponding response information from six immune therapy datasets to evaluate the predictive potential of SRRS for immune therapy responses in CRC patients.The area under the curve (AUC) values for each cohort were calculated using the ‘ROCR’ (35) (v.1.0-11) R package. AUC values were visualized using the ‘pROC’ (36) (v.1.18.0) R package, aiding in the visual interpretation of the model’s performance.

### 2.8 Single-cell data analysis

The count matrix of CRC single-cell data (GSE188711) was imported into the ‘Seurat’ (37) (v.4.2.3) R package. Low-quality cells were identified and excluded based on the following criteria (1): nCount_RNA <1000 or nCount_RNA >20000, and (2) percent.mt > 5. A total of 19,382 high-quality cells were selected for subsequent analyses. Following the Seurat tutorial, we carried out data normalization, cluster identification, and visualization. We manually annotated cell clusters based on previously reported markers. The SRRS values were calculated using the algorithm mentioned above. The t-SNE of single-cell RNA-seq profiles illustrated the annotated cell types and SRRS expression. To identify different pathways in CAFs (Cancer-Associated Fibroblasts) between the high-SRRS and low-SRRS groups, we used the ‘msigdbr’ R package (v.7.5.1) to get human-related pathways. We refined each gene set to have only unique genes and removed genes linked to multiple pathways. Most gene sets kept over 70% of their genes. Then, using the ‘GSVA’ package (v.1.46.0), we determined the pathway activity for each cell. With this data, we analyzed the differences between the two groups using the ‘limma’ R package. To evaluate cell-to-cell communication within the stromal clusters, we conducted the analysis using the ‘CellChat’ (38) R package (version 1.6.1). This provided insight into the incoming communication patterns of the target cells and outgoing communication patterns of the secreting cells.

### 2.9 Messenger RNA expression-based stemness index calculation

The transcriptional mRNAsi of each CRC sample (ranges from zero to one) was computed following the method of Malta et al. using one-class logistic regression machine-learning algorithm (OCLR) based on pluripotent stem cell samples, which strongly correlated with stem cell features and could be applied for cell stemness predictions (39).

### 2.10 Identification of specific chemotherapeutic drugs associated with SRRS

To identify chemotherapeutic drugs uniquely associated with SRRS, following the exclusion of ineffective drugs, we commenced by extracting a dataset comprising 179 distinct drugs from the Genomics of Drug Sensitivity in Cancer (40) (GDSC) database. These drugs were primed for utilization in predicting chemotherapeutic drug sensitivity. We proceeded by employing ‘OncoPredict’ (41) (v.0.2), a specialized R package, to extrapolate each patient’s sensitivity towards the selected drugs, using a ridge regression model operating on a gene expression matrix. This enabled us to draw a comparison of the forecasted drug sensitivities between high-SRRS and low-SRRS groups and pinpoint drugs with a statistically significant variance (|log2FC|>0.4, p<0.05) in sensitivity in each groups.Supplementing this, we ventured into computational drug discovery *via* the Connectivity Map (CMap) database (42). Guided by the principle of “signature reversal”, our aim was to detect drugs that could induce a reversal in gene expression patterns intricately linked to SRRS. We selected the top 100 genes that showcased significant differential expression between the high-SRRS and low-SRRS groups, subjecting them to CMap analysis. The resultant CMap scores, with lower scores denoting a stronger disruptive potential, permitted us to identify a selection of compounds that antagonize the gene expression patterns peculiar to SRRS.

### 2.11 Cell culture and siRNA transfection

The CRC cell line HCT116 was purchased from the American Type Culture Collection (ATCC, Rockville, MD, USA). HCT116 cells were cultured in McCoy’s 5A Medium. All cells were supplemented with 10% fetal bovine serum and 1% penicillin-streptomycin and cultured in a 5% CO2 incubator at 37°C. Small interfering RNA (siRNA) against WRN (si-WRN) and LIMK1 (si-LIMK1), as well as the corresponding negative control (NC), were synthesized by RiboBio (Guangzhou, China), and the transfection of siRNA into CRC cells was performed according to the manufacturer’s protocol. After transfection for 72 hours, the gene knockdown effect was validated using qRT-PCR. The sequences of siRNAs were as follows: si-WRN (sense 5’–3’: GTAGAAGTTTCTCGGTATA; si-LIMK1 (sense 5’–3’): TGGCAAGCGTGGACTTTCA.

### 2.12 RNA extraction and quantitative real-time RT-PCR

Total RNA was extracted from HCT116 cell line using TRIzol reagent (Invitrogen, Carlsbad, CA). cDNA was reverse-transcribed from 1 µg of RNA using an SYBR^®^ Prime ScriptTM RT-PCR kit (Takara Biochemicals, Tokyo, Japan), and quantitative PCR was performed using SYBR Select Master Mix (Roche, Switzerland) and gene-specific primers on an ABI PRISM^®^ 7500HT Real-Time PCR System. The thermal cycling conditions were as follows: an initial step at 95°C for 15 s followed by 40 cycles of 95°C for 5 s and 60°C for 30 s. Each experiment was performed in a 20-µl reaction volume containing 10 µl of SYBR^®^ Prime Ex TaqTM II (2×), 1 µl of forward primer and reverse primer (10 µM each), 2 µl of cDNA, and 7 µl of H2O. β-Actin was used as an internal control. The quantification of the mRNA was calculated using the comparative Ct (the threshold cycle) method according to the following formula: Ratio = 2−ΔΔCT = 2−[ΔCt(sample)-ΔCt(calibrator)], where ΔCt is equal to the Ct of the target gene minus the Ct of the endogenous control gene (β-actin). The primers were as follows: WRN(F:5’-CACAGCAGCGGAAATGTCCT-3’;R:3’-GAGCAATCACTAGCATCGTAACT-5’);LIMK1(F:5’-CAAGGGACTGGTTATGGTGGC-3’;R:3’-CCCCGTCACCGATAAAGGTC-5’).

### 2.13 SA β-gal staining

Senescence-associated β-galactosidase (SA β-gal) activity was measured using a β-gal staining kit (Biolab, Beijing) at pH 6.0, following the manufacturer’s instructions. Briefly, the cells were washed with phosphate-buffered saline (PBS), fixed with 1 ml of fixative solution for 10-15 minutes at room temperature, and then incubated overnight at 37°C with the staining solution mix. The cells were observed under a microscope to assess the level of cellular senescence based on the presence of green coloration.

### 2.14 Colony formation assay

Transfected CRC cells were seeded into 6-well plates at a density of 100–200 cells per well and incubated for 2 weeks. The cells were fixed and stained in a dye solution containing 0.1% crystal violet and 100% methanol. The number of colonies was subsequently counted and analyzed.

### 2.15 Cell proliferation

The RTCA xCELLigence system (ACEA Biosciences Inc., The Netherlands) was used to measure cell proliferation in real-time. CRC cells were placed at a density of 4000–8000/well, and E-plates were then transferred to the RTCA instrument for automated real-time monitoring under standard incubator conditions. Cell proliferation was monitored every 30 min. After 72 h, the measurement was stopped, and the results were analyzed using RTCA software and the results were analyzed after an additional 24 h.

### 2.16 Tumorsphere formation assay

HCT116 cells (1 × 10^3^ cells/well) were seeded into an ultralow-attachment 96-well plate with 200ul of sphere-culturing medium containing serum-free DMEM/F12 medium supplemented with human re- combinant EGF (20 ng/ml), human recombinant basic fibroblast growth factor (10 ng/ml), insulin (4 ug/ml), the optimized serum- free supplement B27, penicillin (500 U/ml), and streptomycin (500ug/ml). Tumorspheres were observed and photographed under microscope after 3–5 days of culture.

### 2.17 Statistical analysis

R software (version 4.2.3) was adopted for statistical analysis. Prior to any parametric statistical tests, the normality of data distribution for each group was assessed using the Shapiro-Wilk test, complemented by visual inspections of histograms. Before conducting ANOVA, the assumption of homogeneity of variances across groups was verified using Levene’s test. For comparisons between two groups, unpaired two-tailed t-tests were applied when data met the assumptions of normality and homogeneity of variances; otherwise, the Wilcoxon rank-sum test was employed as a non-parametric alternative. One-way analysis of variance (ANOVA) with Tukey’s multiple comparisons tests were used for multiple group comparisons. The relationships between variables were estimated with Pearson’s or Spearman’s test. Statistical significance was set at p < 0.05 (*p < 0.05, **p < 0.01, and ***p < 0.001).

## 3 Results

### 3.1 Construct the SRRS model and estimate the senescence status

**Figure 1:**
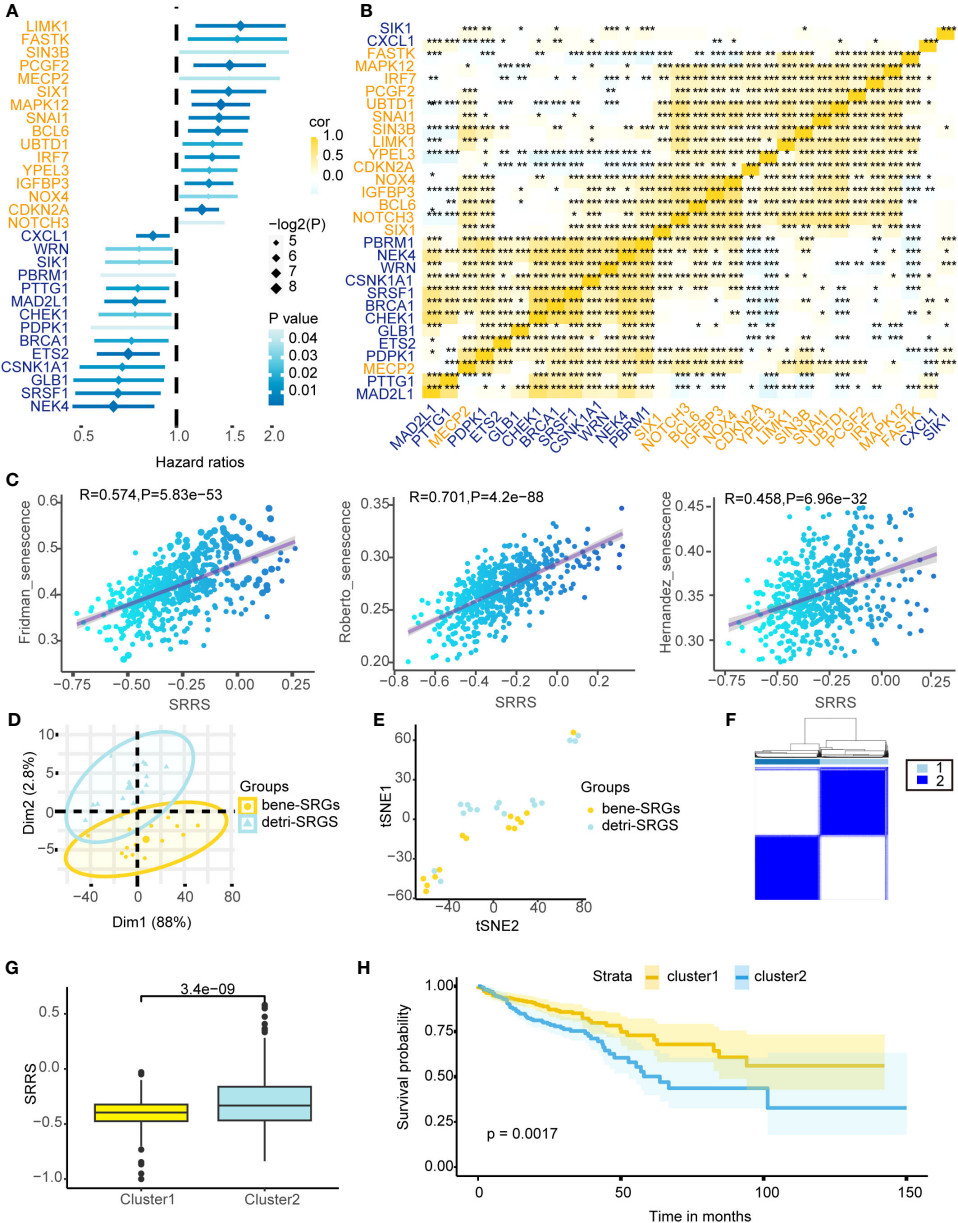
Depicting the prognostic implications of 30 SRGs in TCGA-CRC through survival analysis, correlation mapping, and clustering. **(A)** Univariate Cox regression analysis of overall survival based on gene expression for 30 prognostic-related SRGs in TCGA-CRC cohort. **(B)** Heatmap shows a positive (yellow) and negative (blue) correlation among 30 prognostic-related SRGs in TCGA-CRC cohort. *P < 0.05, **P < 0.01, and ***P < 0.001, as determined by the Spearman correlation analysis. **(C)** Pearson correlation analysis of SRRS and ssGSEA scores for three cellular senescence gene sets. PCA analysis **(D)** and t-SNE analysis **(E)** were performed on the bene-SRGs and detri-SRGS. **(F)** TCGA-CRC samples were divided into two clusters using Consensus clustering based on 30 prognostic-related SRGs. **(G)** Differences in the SRRS between Cluster1 (yellow) and Cluster2 (blue). **(H)** Kaplan-Meier curves compare overall survival between two clusters, Cluster1 (yellow) and Cluster2 (blue), in the TCGA-CRC cohort.

Cellular senescence exhibits a dichotomous nature in colorectal cancer. We identified 279 SRGs (**Supplemental Table 4**) from the CellAge database, employing univariate Cox proportional hazards regression for CRC prognosis prediction, ultimately pinpointing 14 bene-SRGs and 16 detri-SRGS (**Figure 1A**). Expression patterns of these 30 SRGs were thoroughly investigated, and we calculated the pairwise correlation of their expression levels in colorectal cancer, thereby identifying two clusters of SRG correlations: bene-SRGs showed a strong positive correlation with other bene-SRGs and a negative correlation with detri-SRGs, and vice versa (**Figure 1B**). Utilizing PCA and tSNE analyses, we discerned that bene-SRGs and detri-SRGS occupied distinct regions within the CRC landscape (**Figures 1D, E**), suggesting potential functional differences or independence. Accordingly, we employed the ssGSEA to compute the enrichment scores (ES) for bene-SRGs and detri-SRGS, defining these as indicative of the positive and negative components of CRC cell senescence. We further developed SRRS, defined as SRRS = ssGSEA_Score (detri-SRGs) - ssGSEA_Score (bene-SRGs). By performing ssGSEA on three known cellular senescence gene sets, we observed a strong correlation between their ssGSEA scores and SRRS, suggesting the potential of SRRS as a biomarker for assessing the level of cellular senescence in CRC patients (**Figure 1C**). By applying a Consensus Clustering algorithm (43), we classified 589 TCGA-CRC samples into two clusters based on the expression profiles of the 30 SRGs, resulting in an optimal k value of 2. Of these, 303 CRC patients were categorized as Cluster 1, and the remaining 286 as Cluster 2 (**Figure 1F**). In prognostic analyses, patients in Cluster 1 displayed a survival advantage over those in Cluster 2 (**Figure 1H**; P=0.0017). Furthermore, Cluster 1 exhibited a favorable prognosis with a significantly lower SRRS compared to Cluster 2 (**Figure 1G**; p=3.4e-09), suggesting SRRS as a promising prognostic marker reflective of cellular senescence levels in CRC.

### 3.2 SRRS has a good predictive performance in the prognosis of CRC

**Figure 2:**
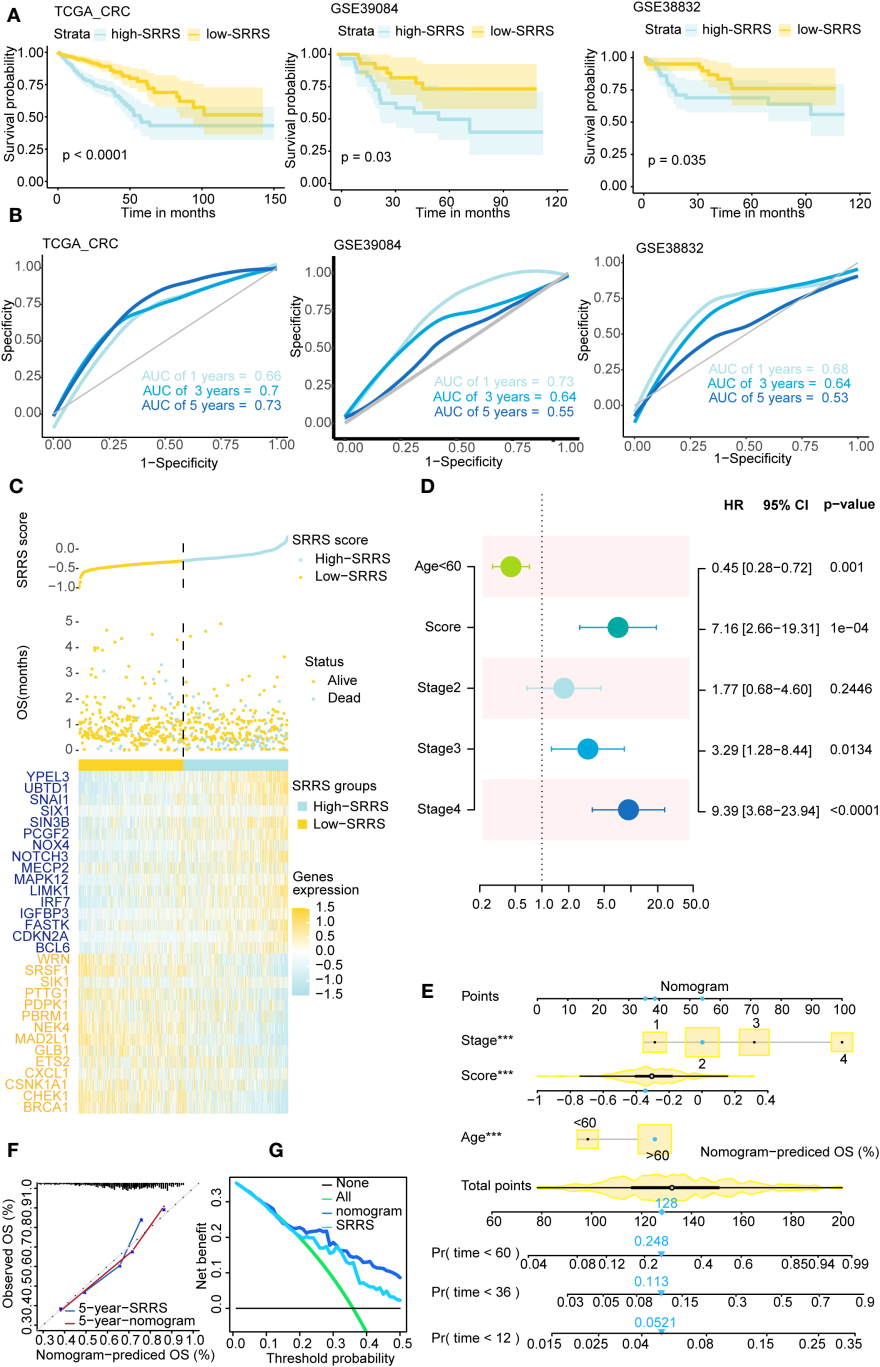
Survival analysis and prediction based on SRRS across different CRC datasets. **(A)** Kaplan-Meier curves compare overall survival between two groups, low-SRRS group (yellow) and high-SRRS group (blue), in 3 CRC datasets, training set TCGA-CRC (p<0.0001), test set GSE39084 (p=0.03), and test set GSE38832 (p=0.0035). **(B)** ROC curve of 1-, 3-, and 5-year survival were also shown in each cohort. **(C)** The distribution of survival status and SRRS in TCGA-CRC. The patients were ordered according to the SRRS, shown in the up panel, and the survival status of each patient with a different SRRS was shown in the middle panel. The SRRS model gene expression value has presented in the lower panel. **(D)** Multivariate Cox regression analysis shows clinicopathological parameters associated with OS among CRC subjects in the TCGA-CRC dataset. **(E)** Nomogram with age, clinical stage, SRRS for predicting 1-year, 3-year, and 5-year OS among CRC patients. **(F)** Calibration curves for the 5-year time points. **(G)** Decision curve analysis shows predicted 5-year OS among TCGA-CRC patients on the basis of the nomogram, SRRS. ***, p < 0.001.

CRC patients were divided into low-SRRS and high-SRRS groups based on the median SRRS. Kaplan-Meier survival curves showed that CRC patients in the low-SRRS group had better clinical outcomes than those in the high-SRRS group: training set TCGA (p<0.0001), test set GSE39084 (p=0.03), GSE38832 (p=0.0035), GSE17536 (p=0.047) and GSE17538 (p=0.0026) (**Figure 2A**; **Supplemental Figure 1B**). Additionally, the receiver operating characteristic (ROC) curves demonstrated a high degree of accuracy of SRRS in predicting overall survival (OS) in the training and test sets (**Figure 2B**; **Supplemental Figure 1C**). Furthermore, we have identified three cellular senescence-related signatures for CRC as proposed by Dai et al., Lv et al., and Yue et al (44–46). Using the TCGA-CRC database, we compared the time-dependent AUC values of SRRS with these signatures, and found our signature to exhibit remarkable performance (**Supplemental Figure 1F**). SRRS and life status scatter plots are also presented in the TCGA-CRC dataset (**Figure 2C**). Following adjustment for clinical and pathological parameters (Exclude CRC patients with missing values in the clinical data.) such as age, gender, clinical stage, T staging, N staging, and M staging (**Supplemental Figure 1A**), multivariable Cox regression analysis was conducted using TCGA data to further ascertain whether SRRS accurately predicted the prognosis of CRC patients. The results showed that SRRS, age, and clinical stage were independent prognostic factors for OS in the training data set (**Figure 2D**). To construct a practical clinical assessment tool to enhance the prediction accuracy of individual CRC OS, we developed a nomogram incorporating clinical stage, age and SRRS to predict the 1-year, 3-year, and 5-year OS probabilities in the TCGA-CRC dataset (**Figure 2E**). As demonstrated by the calibration plot, the nomogram performed better in predicting 1-year, 3-year and 5-year OS than using SRRS alone (**Figure 2F**; **Supplemental Figure 1D**). In the decision curve analysis (47) (DCA) for the corresponding 1-year, 3-year, and 5-year OS, the nomogram exhibited improved net benefits and a broader range of threshold probabilities (**Figure 2G** and **Supplemental Figure 1E**).

### 3.3 High SRRS associated with increased multi-site mutations and differential methylation patterns in CRC

**Figure 3:**
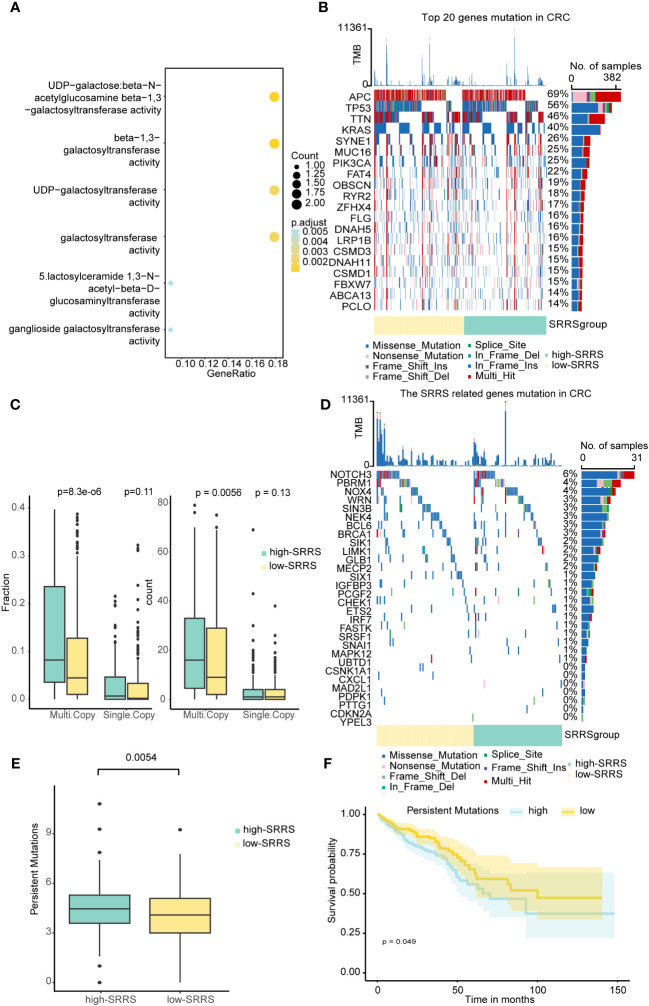
Epigenetic and genetic differences between the high-SRRS and low-SRRS groups. **(A)** Enrichment analysis of gene ontology (GO) terms for Genes mapped to DMPs. Heatmap showing the top 20 mutation events **(B)** and SRRS model gene **(D)** for individual TCGA-CRC patients in the high-SRRS and low-SRRS groups, respectively. Bar plots in the top panel represent the TMB of individual patients. Statistical graph of mutation events for each gene is shown in the left panel. Colors are variant classifications. **(C)** Differences in single mutations and multiple mutations between the high-SRRS and low-SRRS groups. **(E)** Differences in the persistent mutations between the high-SRRS group and low-SRRS group. **(F)** Kaplan-Meier curves compare overall survival between the high and low persistent mutation groups in the TCGA-CRC cohort.

To further explore the potential mechanisms through which SRRS influences the prognosis of CRC patients, we delved into the epigenetic and genetic differences between the high-SRRS and low-SRRS groups. We first analyzed the global methylation data available for the TCGA-CRC cohort (using Illumina 450k chip data) and identified 146 differentially methylated positions among the patients. These differentially methylated positions were mapped to 14 genes (**Supplemental Table 5**) and were found to be predominantly enriched in pathways related to beta-1,3-galactosyltransferase (**Figure 3A**), a classical pathway in cellular senescence. This provides methylation-level evidence for the reliability of SRRS in characterizing cellular senescence. Next, we performed a mutation analysis using the R ‘maftools’ (v.2.14.0) package. We found no significant differences in the top 20 gene mutations between the high-SRRS group and the low-SRRS group (**Figure 3B**). However, we noticed that the 30 genes that constitute the SRRS model manifested more multi-mutations in the high-SRRS group (**Figure 3D**). Upon further investigation of the mutation data, we found that both the Multi-Copy Mutation Count and the Multi-Copy Mutation Fraction were higher in the high-SRRS group than in the low-SRRS group (**Figure 3C**). Furthermore, we investigated the differences in various types of mutations between the high-SRRS and low-SRRS groups, including Microsatellite Instability (MSI), Assessed Mutations, Persistent Mutations, Tumor Mutational Burden (TMB), and Clonal Mutations. Our findings revealed that only Persistent Mutations exhibited a significant difference between the two groups (p=0.0054) (**Figure 3E**; **Supplemental Figure 2A**). Based on the median number of Persistent Mutations, we divided the CRC patients into two groups and found that Persistent Mutations had a significant impact on prognosis (p=0.049) (**Figure 3F**), which might be an important factor influencing the prognostic prediction capability of SRRS. This data provides an in-depth understanding of the genetic and epigenetic differences associated with SRRS and their potential roles in determining CRC prognosis.

### 3.4 SRRS is related to tumor immune infiltration and can predict immunotherapy response

**Figure 4:**
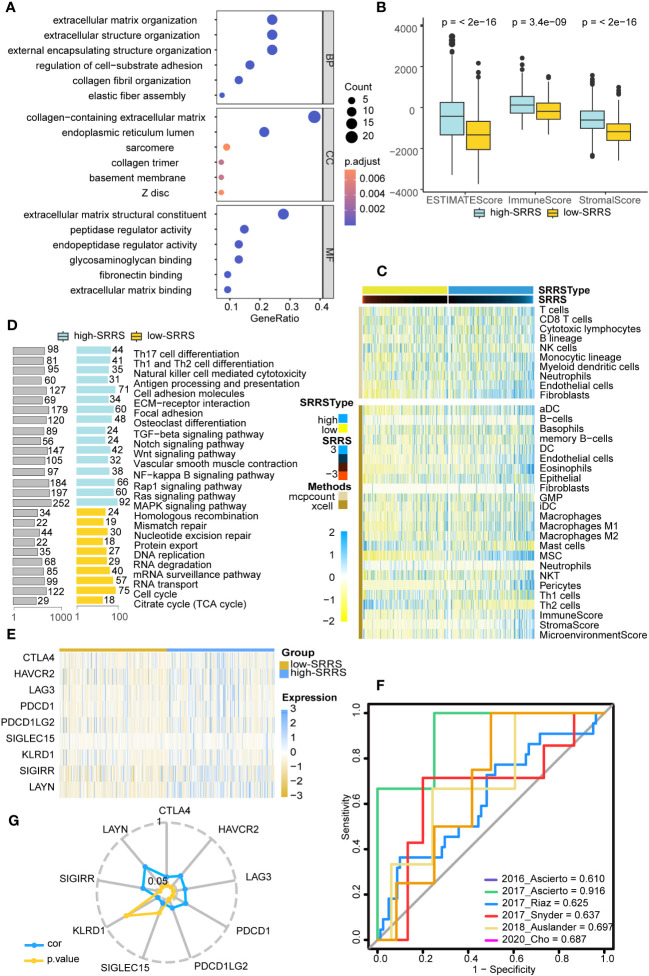
Immune landscape differences between the high-SRRS and low-SRRS groups. **(A)** Enrichment analysis of gene ontology (GO) terms for differential genes between high-SRRS and low-SRRS groups. **(B)** Stromal score, Immune score and ESTIMATE score between high-SRRS and low-SRRS groups. **(C)** The landscape of immune cell infiltration between high-SRRS and low-SRRS groups. **(D)** Enrichment analysis of Kyoto Encyclopedia of Genes and Genomes (KEGG) terms for differential genes between high-SRRS and low-SRRS groups. **(E)** Differences in the expression levels of immune checkpoint genes between high-SRRS and low-SRRS groups. **(F)** Receiver operating characteristic (ROC) curves of the SRRS in distinguishing responders and nonresponders to immunotherapy in six different cohorts. AUCs were calculated by ROC analysis and are labeled in the bottom right. **(G)** Radar plot showing the correlation between SRRS and the expression levels of immune checkpoint genes.

Given that persistent mutations have been reported to sensitize cancer cells to immunotherapies and continuously drive anti-tumor immune responses (20), we suspected differences in immune infiltration between high-SRRS and low-SRRS groups, particularly in light of the observed differences in Persistent Mutations. To investigate this further, we used the R package ‘clusterProfiler’ for GO enrichment analysis and found that most of the enriched pathways were related to the cell matrix and cytoskeleton, potentially reflecting morphological changes in senescence cells (**Figure 4A**). We then conducted GSEA to identify differentially enriched hallmark gene sets between the high-SRRS and low-SRRS groups. We discovered that genes overexpressed in the high-SRRS group were enriched in the NF-kappa B signaling pathway and TGF-beta signaling pathway, consistent with the observed increase in inflammation during senescence. Overexpressed genes in the low-SRRS group were primarily enriched in the Cell cycle and some repair and metabolic pathways, aligning with cell cycle arrest observed during cellular senescence (**Figure 4D**). Moreover, the high-SRRS group was enriched in many pathways related to cell adhesion and proliferation, such as Cell adhesion molecules, ECM-receptor interaction, Focal adhesion, Osteoclast differentiation, Vascular smooth muscle contraction, etc., which might be related to cancer cell proliferation and metastasis. In line with our hypothesis, the high-SRRS group showed significant enrichment in immune gene sets, such as Th17 cell differentiation, Th1 and Th2 cell differentiation, Natural killer cell-mediated cytotoxicity, Antigen processing and presentation etc. (**Supplemental Figure 3C**), suggesting a connection between high SRRS and immune infiltration. Utilizing the ‘estimate’ algorithm based on TCGA-CRC transcriptome data, we calculated Stromal scores, Immune scores, and ESTIMATE scores within malignant tumor tissues. As displayed, both the Stromal and Immune scores were higher in the high-SRRS group compared to the low-SRRS group (**Figure 4B**). Analysis with ‘MCPcount’ and ‘Xcell’ revealed higher levels of most immune cells in the high-SRRS group, except for Th2 cells and mast cells (**Figure 4C**). We speculated that SRRS could predict responses to immunotherapy. After gathering data from six immunotherapy cohorts, we evaluated the predictive capacity of SRRS using the AUC. The AUC scores were 0.610, 0.916, 0.625, 0.637, 0.697, and 0.687, reflecting a robust predictive capacity and validating the potential of SRRS to predict immunotherapy responses (**Figure 4F**; **Supplemental Tables 6–11**). Following this, we explored the relationship between high-SRRS and low-SRRS groups with immune checkpoints. We found that the expression levels of various immune checkpoint genes were higher in the high-SRRS group (**Figure 4E**), possibly suggesting immune cells in the high-SRRS group are likely to exhaust. Correlation analysis of SRRS with the expression levels of various immune checkpoint genes indicated that LAYN(R=0.418) is the most probable potential immune checkpoint (**Figure 4G**). Moreover, we examined the relationships between high-SRRS and low-SRRS groups and major histocompatibility complexes (MHC) as well as T-cell stimulants (**Supplemental Figures 3A, B**). We found that the expression levels of MHC and T-cell stimulants were higher in the high-SRRS group, This may indicate a heightened immune response or increased immune activity in high-SRRS group.

### 3.5 Single-cell analysis reveals stromal cell dominance and potential prognostic factors in high-SRRS CRC

**Figure 5:**
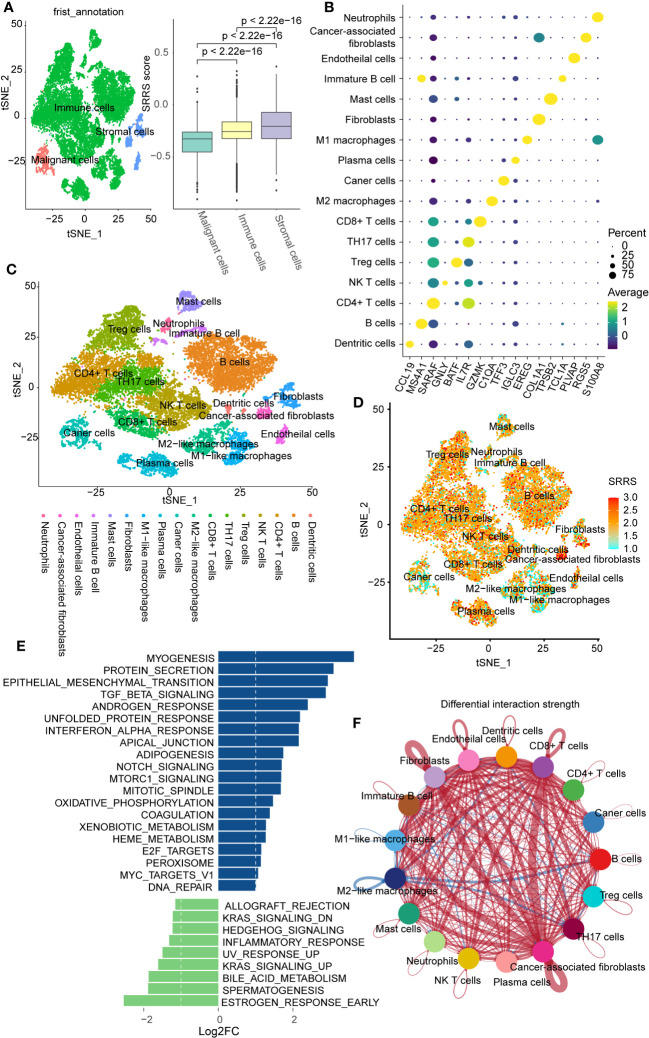
Single-cell transcriptomic profiles in the high-SRRS and low-SRRS groups. **(A)** t-SNE representation of single cells color-coded by major cell type: Malignant, Stromal, and Immune. Bar plot shows SRRS differences. p-values determined by two-sided Wilcoxon test. **(B)** DotPlot for marker genes of each subtype in single cell transcriptomic profiles. **(C)** t-SNE representation of single cells. **(D)** t-SNE plot shows SRRS of whole tissue cells. **(E)** Differences in pathway activities scored per cell by GSVA between the high-SRRS and low-SRRS stromal cell. **(F) **The difference in intercellular communication intensity between the high-SRRS and low-SRRS cells.

To further explore the relationship between SRRS and immunity at the single-cell level, we obtained single-cell sequencing data for colorectal cancer from GSE188711. Following initial cell clustering, we classified cells into immune cells, stromal cells, and malignant cells. Our findings indicated that stromal cells scored higher than immune cells and malignant cells scored lowest (**Figure 5A**). Additional cell clustering (**Figures 5B, C**) revealed that except for Mast cells, Neutrophils, Immature B cells, and Macrophages, other immune cells generally had high SRRS scores, consistent with our previous analyses (**Figure 5D**). Interestingly, we found that cells with the highest SRRS were CAFs (Cancer-Associated Fibroblasts). We employed the ‘GSVA’ R package in conjunction with human gene sets extracted from the ‘msigdbr’ R package to assign pathway activity scores to each CAFs. Subsequently, we analyzed the differential pathways between the low-SRRS and high-SRRS groups using the ‘limma’ R package. The most significant differential pathways between the high-SRRS and low-SRRS groups are MYOGENESIS, PROTEIN_SECRETION, and EPITHELIAL_MESENCHYMAL_TRANSITION (**Figure 5E**). Notably, while the majority of CAFs are derived from resident fibroblasts (48), evidence suggests that other cell types, including tumor cells, can undergo the EMT (epithelial-mesenchymal transition) process and subsequently transform into CAFs (49). The identification of the EMT pathway in our results underscores the possibility of such transformations, especially given that fibroblasts inherently do not undergo EMT. Considering the established role of CAFs in promoting tumor proliferation and the potential of tumor cells that have undergone EMT to facilitate tumor migration (50, 51), we posit that these pathways may be intricately linked to tumor cell proliferation and metastasis. Cell communication analysis revealed that cells in the high-SRRS group had more instances of communication than those in the low-SRRS group, and CAFs were the most frequently communicating with other cells (**Figure 5F**; **Supplemental Figures 4A, B**). This suggests that the potential reason for the poor prognosis in patients in the high-SRRS group could be the proliferation and EMT of CAFs.

### 3.6 Strong negative correlation between SRRS and cell stemness in CRC

**Figure 6:**
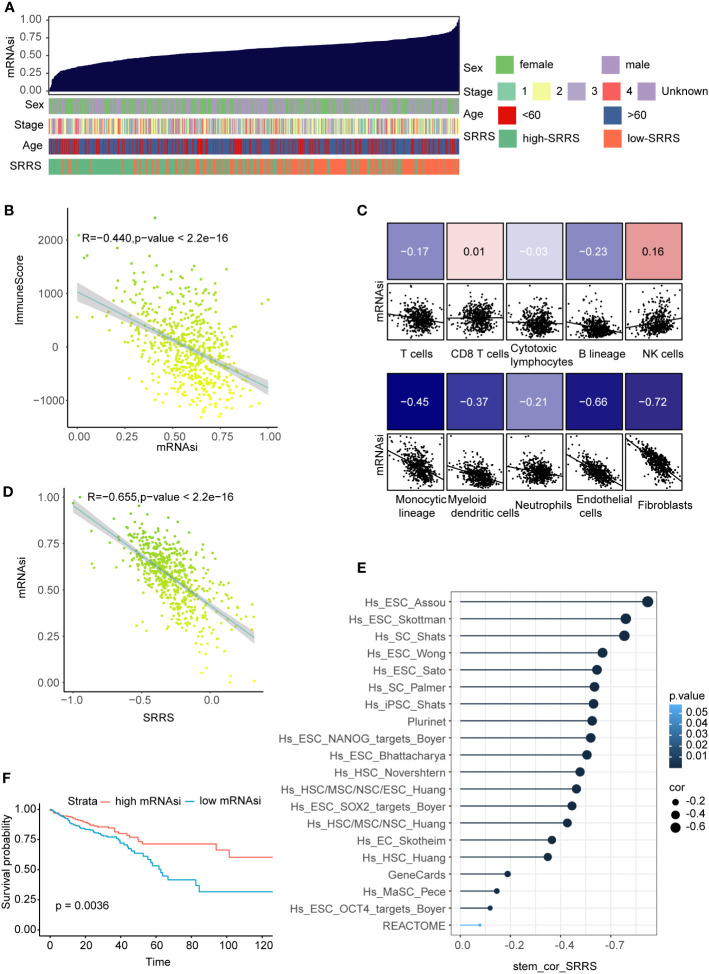
Correlation of SRRS with mRNAsi, immune scores, and overall survival. **(A)** The overview of correlation between mRNAsi and clinical features as well as SRRS. **(B)** Pearson correlation analysis of mRNAsi score and immuneScore. **(C)** Pearson correlation analysis between the abundance of various immune cell components in MCPcounter analysis and the SRRS. **(D)** Pearson correlation analysis between the SRRS and mRNAsi. **(E)** Pearson correlation analysis between ssGSEA scores of the stemness-associated gene sets. **(F)** Kaplan-Meier curves compare overall survival between the high-SRRS group and low-SRRS group.

Prevailing evidence suggests an inverse correlation between immune infiltration and cell stemness (52, 53). Harnessing the one-class logistic regression machine-learning algorithm (OCLR) on TCGA-CRC, we computed the mRNAsi for each sample as a surrogate marker of cell stemness. In evaluating mRNAsi across a gradient from low (left) to high (right), a noteworthy aggregation of high-SRRS predominantly within regions of low mRNAsi was observed (**Figure 6A**). Furthermore, a notable inverse correlation(R=-0.440) emerged between mRNAsi and ImmuneScore derived from estimate analysis (**Figure 6B**). Probing deeper into the relationships between mRNAsi and various immune components—calculated with the ‘MCPcounter’ R package—we discovered strong negative correlations with endothelial cells(R=-0.66, p<0.05), and fibroblasts(R=-0.73, p<0.05) (**Figure 6C**). Given our preceding observations, highlighting elevated SRRS in stromal cells and intensified immune infiltration in the high-SRRS cohort, we hypothesized a potential association between SRRS and CRC cell stemness. A subsequent correlation analysis between SRRS and mRNAsi indeed unveiled a robust negative correlation (**Figure 6D**), intimating SRRS’s capability to predict CRC cell stemness. To further validate this, we compiled 20 stemness-associated gene sets from prior research, conducting ssGSEA for each within the TCGA-CRC context, and followed with a correlation analysis of the ssGSEA scores and SRRS. A consistent and predominantly strong negative correlation(R=-0.655) was discerned (**Figure 6E**), solidifying SRRS’s predictive ability regarding CRC cell stemness. Importantly, Kaplan-Meier survival analyses revealed that CRC patients with high mRNAsi demonstrated superior OS compared to their low mRNAsi counterparts (**Figure 6F**).

### 3.7 Identification of specific chemotherapeutic drugs associated with SRRS

**Figure 7:**
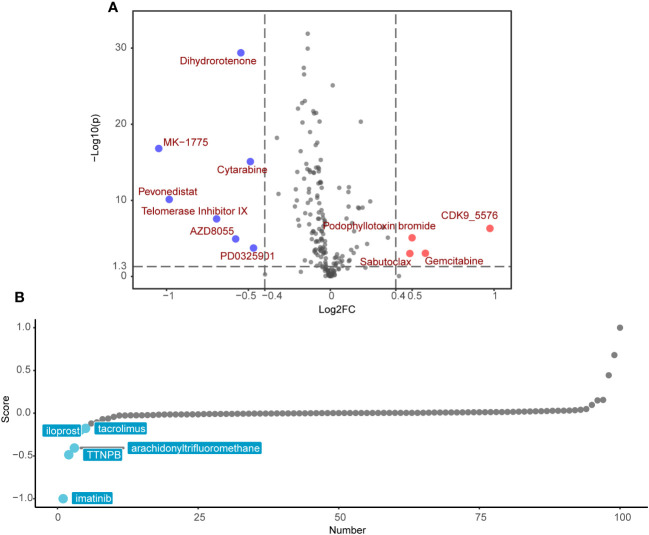
Potential drug candidates for different SRRS groups. **(A)** Drug candidates with potential therapeutic effect for the low-SRRS group or the high-SRRS group. **(B)** Candidate drugs with potential therapeutic effect to reverse SRRS-associated gene expression patterns.

Following the exclusion of ineffective drugs (those presenting NA values in more than 20% of the samples), we obtained a collection of 179 chemotherapeutic agents from the GDSC2 drug genomic database for drug sensitivity prediction. Using the ‘OncoPredict’ R package, we employed a ridge regression model premised on gene expression matrices to predict drug sensitivity for each patient. We then explored the differences in predicted drug sensitivity between the high-SRRS and low-SRRS groups. This analytical approach identified 11 drugs that were statistically significant (|log2FC|>0.4, p<0.05). Among them, the low-SRRS group presented seven drugs, including MK−1775, Pevonedistat, Telomerase Inhibitor IX, Dihydrorotenone, AZD8055, Cytarabine and PD0325901, while the high-SRRS group identified four drugs, such as CDK9_5576, Gemcitabine, Sabutoclax and Podophyllotoxin bromide (**Figure 7A**). Guided by the principle of “signature reversal,” we employed CMap data for computational drug discovery, intending to pinpoint drugs capable of reversing SRRS-associated gene expression patterns. We selected the top 100 differentially expressed genes between the high-SRRS and low-SRRS groups for CMap analysis, and chose the top five lowest drugs as potential drugs capable of reversing the SRRS-related gene expression pattern. The results showed that the compounds iloprost, tacrolimus, TTNPB, arachidonyltrifluoromethane, and imatinib are potential drugs that can reverse the SRRS-related gene expression pattern (**Figure 7B**). It is noteworthy that both the GDSC2-predicted drug MK-1775 and the Cmap-predicted drug imatinib are tyrosine kinase inhibitors (TKIs). Tyrosine kinases play a pivotal role in DNA damage response (54). Inhibiting these kinases can make cancer cells more susceptible to being killed during treatment, especially when used in combination with other anticancer drugs. The fact that both methods pointed towards TKIs underscores the potential relevance of these drugs in the context of CRC (55).

### 3.8 Unveiling the functional influence of key SRRS genes, LIMK1 and WRN, on CRC cell phenotypes

**Figure 8:**
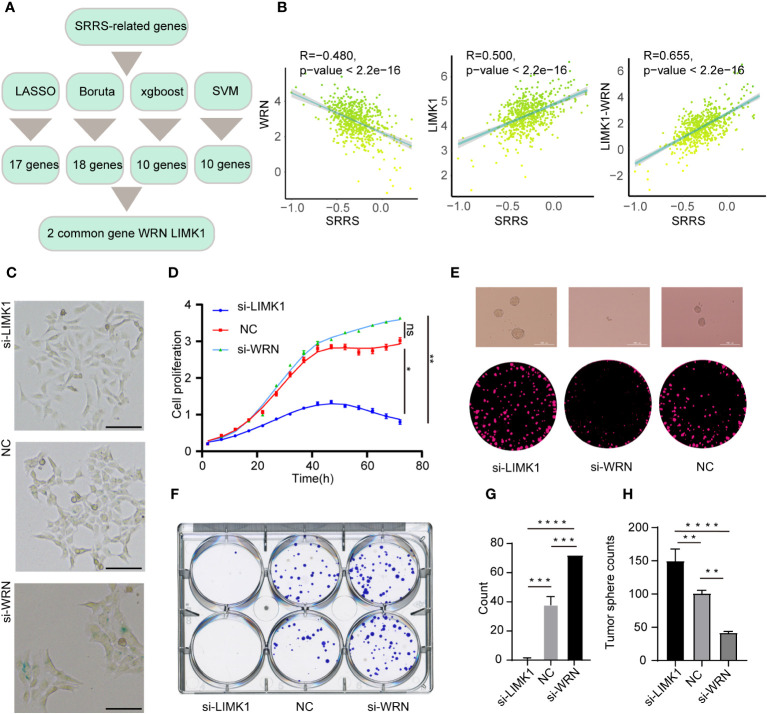
Strategies for key gene selection in SRRS signature, gene knockdown effects on cell proliferation and formation **(A)** Overview of the strategies employed for selecting key genes in the SRRS signature to predict Overall Survival (OS) of CRC patients in the TCGA cohort. **(B)** Pearson correlation analysis illustrating the relationships between SRRS and the expression levels of WRN and LIMK1, and the difference between these expressions. **(C)** β-Galactosidase staining of HCT-116 cells following knockdown of LIMK1 and WRN genes, compared with negative control cells. Scale Bar: 100μm. **(D)** Proliferation analysis of HCT-116 cells within the LIMK1 and WRN gene knockdown cell lines, along with the negative control line. **(F, G)** Examination of colony formation capabilities in LIMK1 and WRN gene knockdown cells, in contrast to negative control cells. **(E)** Representative images and **(H)** counts of cell spheres for LIMK1 and WRN gene knockdown cells, in comparison with negative control cells. *, p < 0.05; **, p < 0.01; ***, p < 0.001 ****, p < 0.0001; "ns" for "not significant".

A significant challenge for the clinical application of the SRRS model resides in its extensive genetic composition. To enhance the feasibility of SRRS gene signatures in prognostic evaluations, we leveraged four machine learning algorithms to distill key features from the comprehensive gene cohort of the SRRS model. We deployed the least absolute shrinkage and selection operator (LASSO), support vector machine-recursive feature elimination (SVM-RFE), random forest and boruta (RFB), and extreme gradient boosting (XGBoost) methodologies, identifying 17, 10, 18, and 10 pertinent genes (**Supplemental Table 12**), respectively (**Figure 8A**). Commonality emerged in two genes, WRN from bene-SRGs and LIMK1 from detri-SRGs, which we thus deemed as the hub genes of the SRRS model. We hypothesized that the differential expression of LIMK1 and WRN could symbolize the SRRS. Our correlation analysis substantiated this, revealing a robust positive correlation (R=0.655) between LIMK1-WRN and SRRS (**Figure 8B**). In pursuit of understanding the functions of these two vital SRRS genes within CRC cells, we established si-LIMK1 and si-WRN CRC cell lines through siRNA transfection against LIMK1 and WRN, respectively. Intriguingly, β-galactosidase staining demonstrated that si-LIMK1 cells showed a lighter staining compared to the negative control (NC), whereas si-WRN cells were deeply stained (**Figure 8C**), indicating that LIMK1 suppression could curtail cellular senescence, while WRN reduction could instigate it. Cell proliferation (**Figure 8D**) and colony formation (**Figures 8F, G**) assays yielded further insights: compared to NC, si-LIMK1 cells manifested a deceleration in proliferation, whereas si-WRN CRC cells proliferated more swiftly. This insinuates that LIMK1 knockdown could curtail tumor cell proliferation, while WRN knockdown could stimulate it. Finally, we gauged the stemness phenotype of the LIMK1 and WRN knockdown cells *via* sphere formation assays. The results unveiled a significant amplification in both sphere number and size in si-LIMK1 cells compared to NC, with a concurrent diminution observed in si-WRN cells (**Figures 8E, H**). In summary, our functional experiments demonstrated that inhibiting LIMK1 reduces cellular senescence and inhibits tumor cell proliferation, while reducing WRN has the opposite effect, indicating their potential as therapeutic targets.

## 4 Discussion

The study offers comprehensive insight into the relevance of the SRRS in CRC, unraveling details of its impacts on cellular senescence, stemness, immune infiltration, stromal cell activity and drug sensitivity. Strong associations have been identified between higher SRRS and worse prognosis in CRC patients, bolstering the credibility of SRRS as a potent predictor of patient survival rates.

Our results clarify the possible pathways through which SRRS can affect prognosis in CRC patients, such as its link to increased multi-site mutations and distinctive methylation patterns. These genetic and epigenetic modifications are pivotal determinants of cancer cell biology, manipulating cell proliferation, invasion, and metastasis of the cancer (56–59). Interestingly, the high-SRRS group exhibits a higher number of persistent mutations, substantially affecting patient prognosis. The ongoing nature of these mutations can stimulate anti-tumor immune responses and increase cancer cells’ sensitivity to the immunotherapy. A hypothesis is formed suggesting that SRRS can forecast immunotherapy responses which has been later confirmed through further investigation.

Connections are also observed between high SRRS and amplified immune cell infiltration, consistent with the high stromal scores and immune scores identified in the high-SRRS group. Elements such as immune cell infiltration and stromal cell activity are vital components of the tumor microenvironment, exerting significant influences on tumor progression and patient prognosis (60–63). Additionally, this study pinpoints LAYN as a potential immune checkpoint showing the most significant correlation with SRRS, sparking further inquiry into the function of LAYN in CRC and its potential as a therapeutic target (64). Notably, single-cell RNA analysis shows a preponderance of CAFs in the high-SRRS group. A surge in CAFs in tumors can drive cancer progression and metastasis, implying that proliferation and EMT of CAFs may contribute to the adverse prognosis for patients in the high-SRRS group.

In view of the stemness of tumor cells tending toward forming “cold” tumor (65, 66), a strong inverse correlation between SRRS and cell stemness is also identified from this study, suggesting a role for SRRS in forecasting CRC cell stemness. Understanding this correlation can shed light on the behaviors of cancer cells and possibly guide the identification of novel therapeutic targets for CRC. Notably, specific chemotherapeutic drugs associated with SRRS have been pinpointed in this study. Additionally, exploration was undertaken into the functional influence of two significant SRRS genes, LIMK1 and WRN, on CRC cell phenotypes, potentially assisting in the development of personalized treatment plans for CRC patients based on their SRRS.

In summary, this research contributes to a broader understanding of the role and clinical significance of SRRS in CRC, revealing potential prognostic indicators and therapeutic targets. However, further *in-vitro* and *in-vivo* studies are warranted to validate these findings and to decipher the mechanistic interactions of SRRS in CRC.

## Data availability statement

The original contributions presented in the study are included in the article/**Supplementary Material**. Further inquiries can be directed to the corresponding authors.

## Ethics statement

Ethical approval was not required for the study involving humans in accordance with the local legislation and institutional requirements. Written informed consent to participate in this study was not required from the participants or the participants’ legal guardians/next of kin in accordance with the national legislation and the institutional requirements. Ethical approval was not required for the studies on animals in accordance with the local legislation and institutional requirements because only commercially available established cell lines were used.

## Author contributions

XZ: Data curation, Formal Analysis, Methodology, Software, Validation, Writing – original draft, Writing – review & editing. YH: Data curation, Formal Analysis, Writing – original draft. QL: Formal Analysis, Writing – review & editing. YiZ: Data curation, Formal Analysis, Writing – original draft. YuZ: Formal Analysis, Methodology, Writing – original draft. JH: Formal Analysis, Methodology, Data curation, Writing – original draft. RL: Supervision, Writing – review & editing. XL: Supervision, Writing – review & editing, Conceptualization, Writing – original draft.
