# Supplementary material for: Senescence risk score: a multifaceted prognostic tool predicting outcomes, stemness, and immune responses in colorectal cancer
Source: Front Immunol. 2023 Sep 26;14:1265911. doi: 10.3389/fimmu.2023.1265911 (PMC10566297; doi:10.3389/fimmu.2023.1265911)
Supplement: Supplementary file 1 [file DataSheet_1.docx]

Supplementary Material

Senescence Risk Score (SRRS): A Multifaceted Prognostic Tool Predicting Outcomes, Stemness, and Immune Responses in Colorectal Cancer

Rui Liu*, Xiaoying Luo*, Xiaojun Zhang, Yilan Huang, Xiangyu Tang

*** Correspondence:** Rui Liu:liurui1510193@163.com ; Xiaoying Luo:luoxy@shsci.org

# Supplementary Figures and Tables

## Supplementary Figures


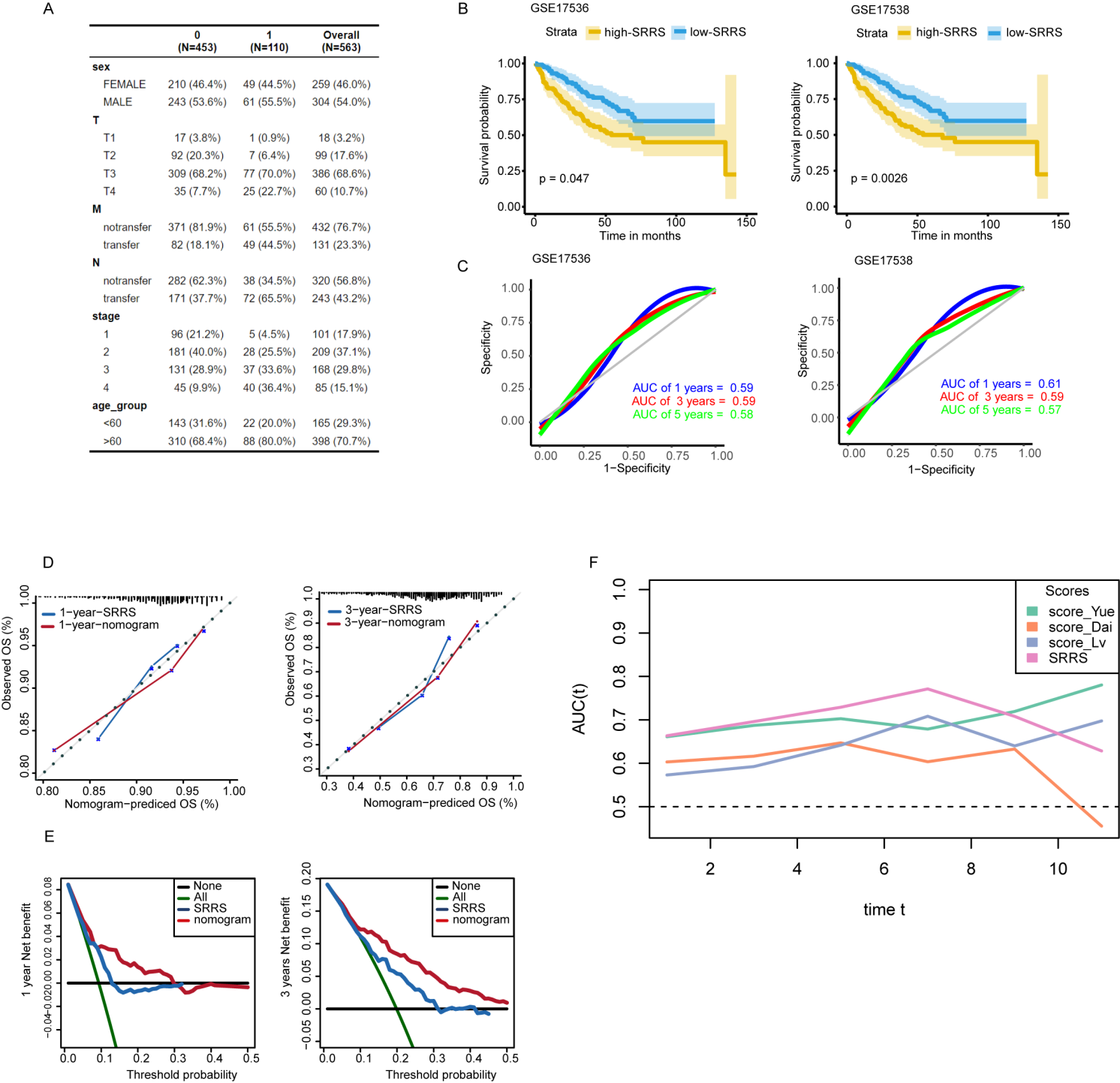


**Supplementary Figure 1.** (**A**) Clinical information table of TCGA-CRC patients. (**B**) Kaplan-Meier curves compare overall survival between two groups, low-SRRS group and high-SRRS group , in test set GSE17536 (p=0.047), and test set GSE17538 (p=0.0026). (**C**) ROC curve of 1-, 3-, and 5-year survival were also shown in GSE17536 and GSE17538. (**D**) Calibration curves for the 1-year and 3-year time points in TCGA-CRC. (**E**) Decision curve analysis shows predicted 1-year OS and 3-year OS among TCGA-CRC patients on the basis of the nomogram, SRRS. (**F**) Comparing the time-dependent AUC values of senescence-related signatures in SRRS with those of three other studies: Dai et al., Lv et al., and Yue et al.


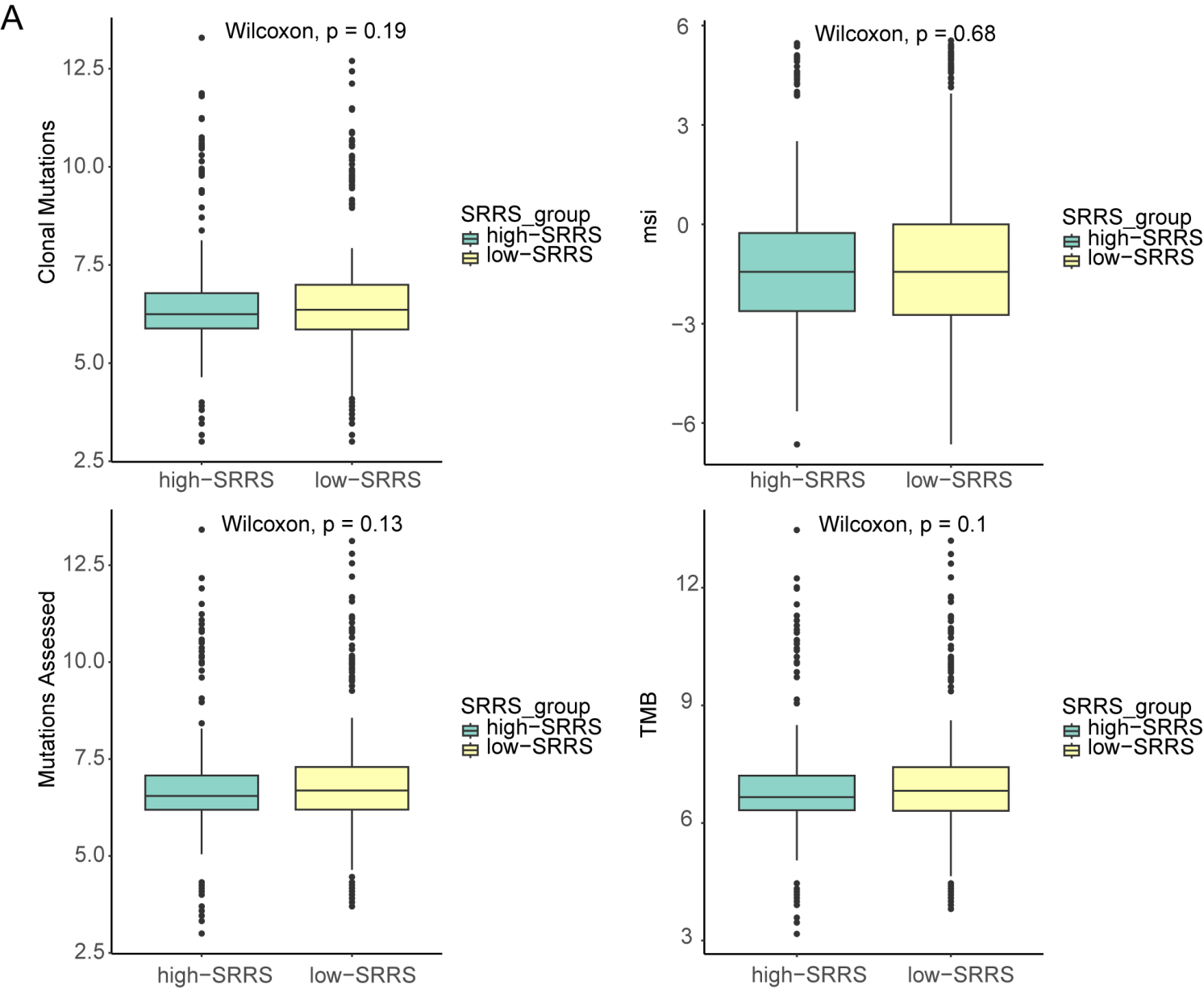


**Supplementary Figure 2**. (**A**) Differences in Clonal Mutations, msi (microsatellite instability), Mutations Assessed and TMB (tumor mutational burden) between the high-SRRS group and low-SRRS group.


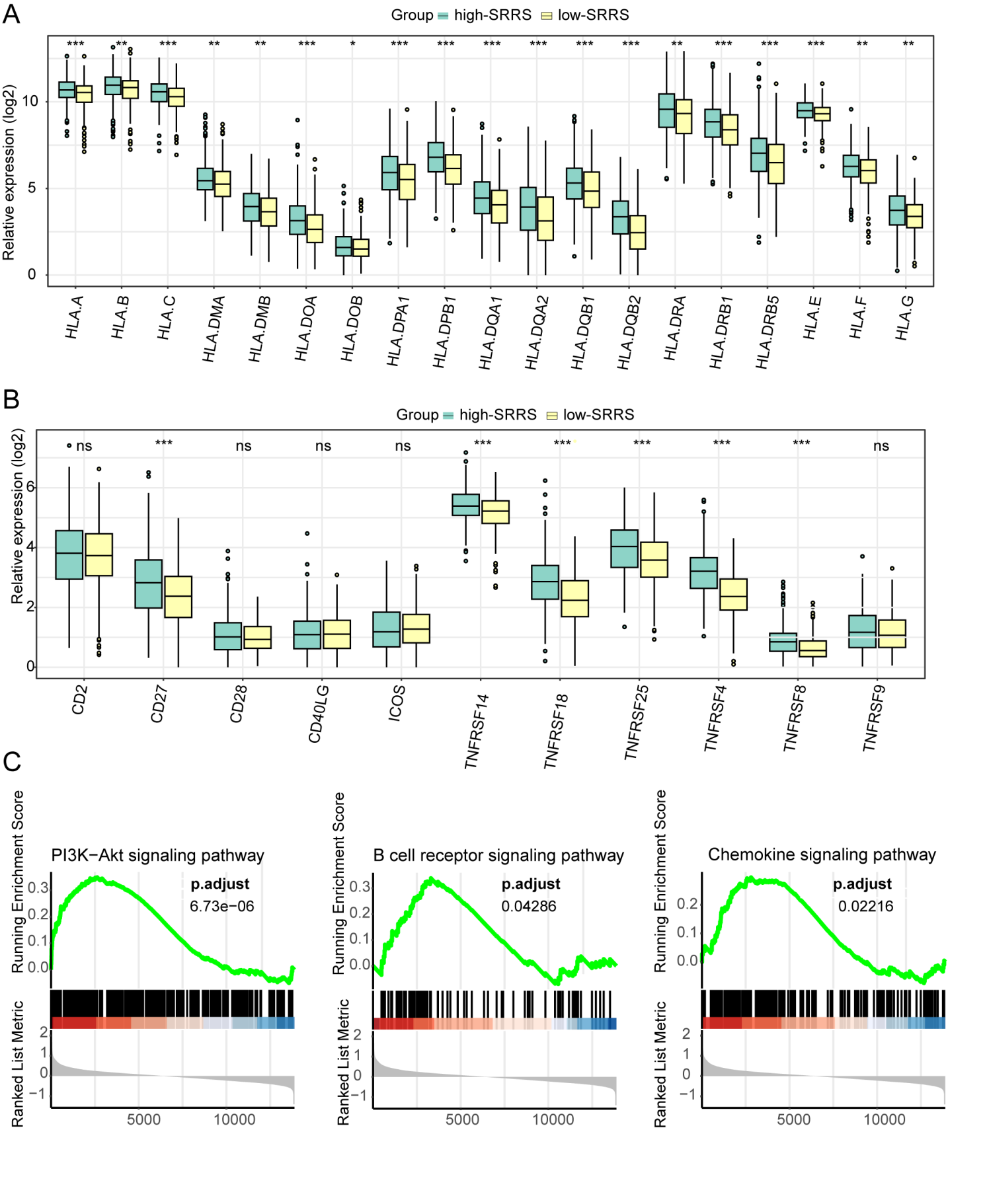


**Supplementary Figure 3.** (**A**) Differences in HLA-related gene expression between the high-SRRS group and the low-SRRS group. (**B**) Differences in T-cell stimulant-related gene expression between the high-SRRS group and the low-SRRS group. (**C**) Differential gene enrichment in immune-related pathways between the high-SRRS group and the low-SRRS group.


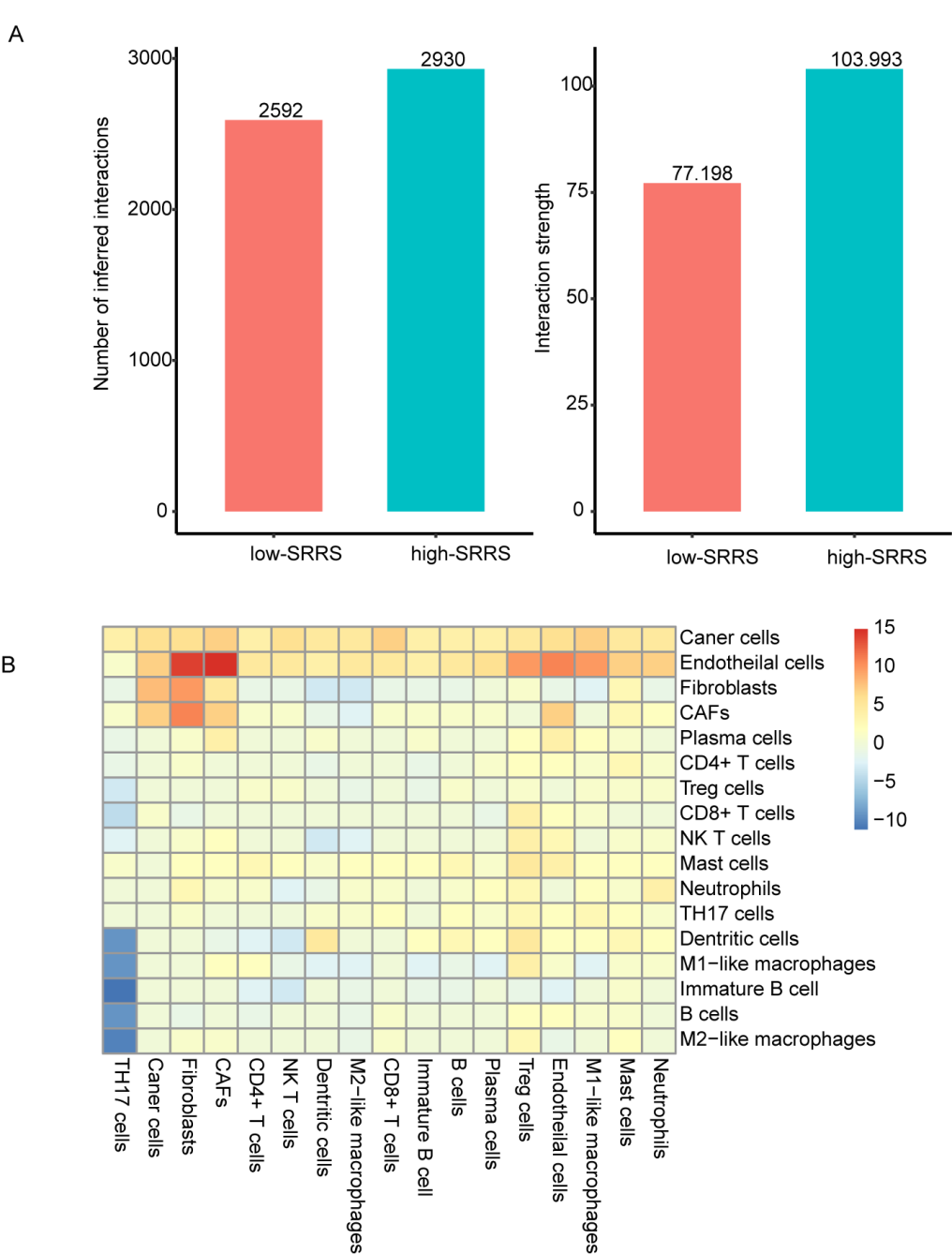


**(1)**
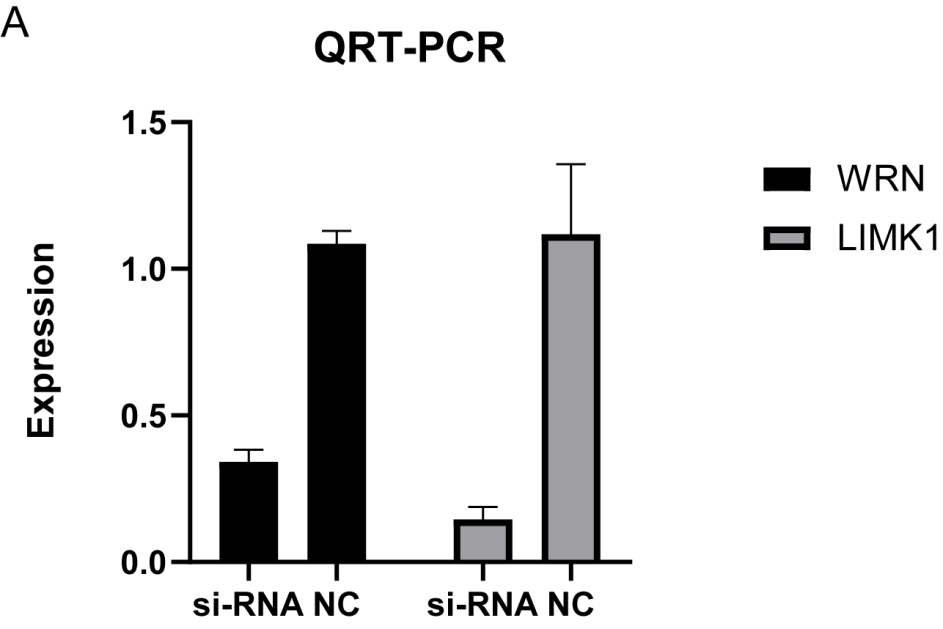


**Supplementary Figure 5**. (**A**) qRT-PCR assessment of WRN expression in si-WRN cells relative to controls, and LIMK1 expression in si-LIMK1 cells relative to controls.

## Supplementary Tables

Refer to the additional supplementary material.

1. Huang L, Jiang S, Shi Y. Tyrosine Kinase Inhibitors for Solid Tumors in the Past 20 years (2001-2020). *J Hematol Oncol* (2020) 13(1):143. doi: 10.1186/s13045-020-00977-0.
